# Marine-Derived Chitosan Nanoparticles Improved the Intestinal Histo-Morphometrical Features in Association with the Health and Immune Response of Grey Mullet (*Liza ramada*)

**DOI:** 10.3390/md18120611

**Published:** 2020-12-01

**Authors:** Mahmoud A.O. Dawood, Mahmoud S. Gewaily, Ali A. Soliman, Mustafa Shukry, Asem A. Amer, Elsayed M. Younis, Abdel-Wahab A. Abdel-Warith, Hien Van Doan, Adel H. Saad, Mohamed Aboubakr, Hany M.R. Abdel-Latif, Sabreen E. Fadl

**Affiliations:** 1 Department of Animal Production, Faculty of Agriculture, Kafrelsheikh University, Kafrelsheikh 33516, Egypt; 2 Department of Anatomy and Embryology, Faculty of Veterinary Medicine, Kafrelsheikh University, Kafrelsheikh 33516, Egypt; msgewaily@vet.kfs.edu.eg; 3 Fish Nutrition Laboratory, Aquaculture Division, National Institute of Oceanography and Fisheries, Alexandria 21556, Egypt; Doctor.ali.soliman@gmail.com; 4 Department of Physiology, Faculty of Veterinary Medicine, Kafrelsheikh University, Kafrelsheikh 33516, Egypt; mostafa.ataa@vet.kfs.edu.eg; 5 Central Laboratory for Aquaculture Research, Abbassa, Sharkia, Sakha Aquaculture Research Unit, Kafrelsheikh 33516, Egypt; asemamer77@yahoo.com; 6 Department of Zoology, College of Science, King Saud University, P.O. Box 2455, Riyadh 11451, Saudi Arabia; emyounis@ksu.edu.sa (E.M.Y.); awarith@ksu.edu.sa (A.-W.A.A.-W.); 7 Department of Animal Production, Faculty of Agriculture, Al-Azhar University, Nasr City, Cairo 11651, Egypt; 8 Department of Animal and Aquatic Sciences, Faculty of Agriculture, Chiang Mai University, Chiang Mai 50200, Thailand; 9 Innoviative Agriculture Research Center, Faculty of Agriculture, Chiang Mai University, Chiang Mai 50200, Thailand; 10 Nutrition and Clinical Nutrition Department, Faculty of Veterinary Medicine, Matrouh University, Matrouh 51744, Egypt; adelhassan1179@mau.edu.eg; 11 Pharmacology Department, Faculty of Veterinary Medicine, Benha University, Moshtohor, Toukh 13736, Egypt; mohamed.aboubakr@fvtm.bu.edu.eg; 12 Department of Poultry and Fish Diseases, Faculty of Veterinary Medicine, Alexandria University, Edfina 22758, Egypt; hmhany@alexu.edu.eg; 13 Biochemistry Department, Faculty of Veterinary Medicine, Matrouh University, Matrouh 51744, Egypt; nourmallak@mau.edu.eg

**Keywords:** nanotechnology, marine chitin, Grey Mullet, intestinal health, immunity

## Abstract

Marine-derived substances are known for their beneficial influences on aquatic animals’ performances and are recommended to improve intestinal health, immunity, and anti-oxidative status. The present study investigates the role of chitosan nanoparticles on the intestinal histo-morphometrical features in association with the health and immune response of Grey Mullet (*Liza ramada*). Chitosan nanoparticles are included in the diets at 0, 0.5, 1, and 2 g/kg and introduced to fish in a successive feeding trial for eight weeks. The final body weight (FBW), weight gain (WG), and specific growth rate (SGR) parameters are significantly increased while feed conversion ratio (FCR) decreases by chitosan nanoparticles compared to the control (*p* < 0.05). The morphometric analysis of the intestines reveals a significant improvement in villus height, villus width, and the number of goblet cells in chitosan-treated groups in a dose-dependent manner. Additionally, there is a positive correlation between the thickness of the enterocyte brush border and the chitosan dose, referring to an increasing absorptive activity. Histologically, the intestinal wall of Grey Mullet consists of four layers; mucosa, sub-mucosa, tunica muscularis (muscular layers), and serosa. The histological examination of the *L. ramada* intestine shows a normal histo-morphology. The epithelial layer of intestinal mucosa is thrown into elongated finger-like projections, the intestinal villi. The values of hemoglobin, hematocrit, red blood cells (RBCs), total protein (TP), albumin, and globulin are significantly increased in fish fed 1, and 2 g/kg of chitosan nanoparticles compared to fish fed 0 and 0.5 g/kg (*p* < 0.05). The highest levels of TP and albumin are observed in fish fed 1 g/kg diet (*p* < 0.05). The lysozyme activity and phagocytic index are significantly enhanced by feeding chitosan nanoparticles at 0.5, 1, and 2 g/kg, whereas the phagocytic activity is improved in fish fed 1 and 2 g/kg (*p* < 0.05). The highest lysozyme activity and phagocytic index are observed in fish fed 1 g/kg. SOD is significantly activated by feeding chitosan nanoparticles at 1 g/kg. Simultaneously, glutathione peroxidase (GPx) and catalase (CAT) activities also are enhanced by feeding chitosan at 1 and 2 g/kg, compared to fish fed 0 and 0.5 g/kg (*p* < 0.05). The highest GPx and CAT activities are observed in fish fed 1 g/kg (*p* < 0.05). Conversely, the malondialdehyde (MDA) levels are decreased by feeding chitosan at 1 and 2 g/kg, with the lowest being in fish fed 1 g/kg (*p* < 0.05). To summarize, the results elucidate that *L. ramada* fed dietary chitosan nanoparticles have a marked growth rate, immune response, and anti-oxidative response. These improvements are attributed to the potential role of chitosan nanoparticles in enhancing intestinal histo-morphometry and intestinal health. These results soundly support the possibility of using chitosan nanoparticles at 1–2 g/kg as a feasible functional supplement for aquatic animals.

## 1. Introduction

The Grey Mullet fish species are classified under the family of (Mugilidae) and can grow in tropical waters with a capacity to live in marine, brackish, and freshwater conditions [1]. These fish species are mainly spread by the countries’ northern coastal areas located on the southern Mediterranean Sea (e.g., Egypt) [2]. Grey Mullet (*Liza ramada*) can be farmed under the Egyptian conditions mostly in polyculture systems due to their high ability to benefit from the natural food cycles in the pond and the ability to utilize the formulated pellets, regardless of their protein content, as well as their high growth rate and their resistance against aquaculture stressors [3,4]. Moreover, fish are expected to suffer from malnutrition and stressful conditions, which induce inflammation in their intestinal health and deteriorate their intestinal immunity [5]. The impairment of their local intestinal immunity is correlated with their general immune response and their ability to resist fish infectious diseases [6]. Traditionally, antibiotics are used to relieve the inflammatory features caused by stress and malnutrition [7]. However, modern food security protocols suggest the necessity for reducing the application of chemotherapies and replace them with friendly alternative substances [8]. There are many feed additives used during the season of farming to improve the performance and wellbeing of aquatic animals [5]. Therefore, marine-derived substances, including chitosan, are proven to exhibit potential effects on the performance of cultured fish [9]. Chitosan is derived from chitin and extracted mainly from crustaceans (e.g., shrimp, crab, and crawfish) [10]. Chitosan has favorable effects, including bioremediation, immunostimulant, and antibacterial properties [11]. Further, dietary chitosan has no toxic influences on the biodegradable and biocompatibility effects [12]. Markedly, chitosans are used as antibacterial agents against pathogenic bacteria in aquaculture. Accordingly, chitosan increases the abundance of beneficial bacteria and decreases the pathogenic ones [13]. The microbial diversity in the fish’s gastrointestinal tract (GIT) strongly participates in regulating intestinal immunity depending on the ratio between the beneficial and pathogenic microorganisms and the composition of the aquafeed nutrients [14]. More specifically, the beneficial bacteria secrete digestive enzymes to digest the nutrients and facilitate their absorption through the intestines of fish, enhancing the feed efficiency and the growth rate [6]. Additionally, anti-oxidative properties are shown in aquatic animals fed diets incorporated with chitosan [15]. Interestingly, chitosan is known for its high resistance against hydrolysis caused by digestive enzymes in the GIT, and the ability to reduce intestinal injury and enhance the expression of tight junction proteins [16]. However, there seems to be no detailed information about using chitosan in aquatic animals to enhance intestinal health and wellbeing.

Nowadays, nanotechnology is widely applied in several sectors due to its functionality and positive effects. It helps minimize particle size and increase surface functionality, showing the maximum possible impact with the lowest possible amounts of a material [17]. Notably, the nanomaterials are applied in the biomedical, food preparation, and aquaculture sectors [18]. Regarding aquaculture-related studies, chitosan nanoparticles are included in the diets of Nile tilapia (*Oreochromis niloticus*) [19,20,21,22], rainbow trout (*Oncorhynchus mykiss*) [23], silver carp (*Hypophthalmichthys molitrix*) [24], and loach (*Misgurnus anguillicaudatus*) [15]. The results show an improved growth rate, feed utilization, immune response, and resistance against pathogens. 

This study aims to investigate the potential role of dietary chitosan nanoparticles on the intestinal histo-morphometry, hemato-biochemical profile, immune response, and anti-oxidative status of Grey Mullet (*L. ramada*). Considering the growth-promoting, anti-inflammatory, anti-oxidative, and immune-enhancing effects of chitosan, it is proposed that chitosan would be a feasible and beneficial feed additive to enhance the GIT histo-morphometrical features of *L. ramada*.

## 2. Results

### 2.1. Growth Performance

Chitosan nanoparticles significantly increased the final body weight (FBW), weight gain (WG), and specific growth rate (SGR) parameters compared to the control (*p* < 0.05). The value of feed conversion ratio (FCR) was reduced in the group of *L. ramada* receiving 0.5, 1, and 2 g/kg of chitosan nanoparticles, with the lowest values being in fish fed 2 g/kg (*p* < 0.05) (Table 1).

### 2.2. Intestinal Histomorphology

The results of the morphometric analysis of the intestines revealed a significant improvement in villus height, villus width, and the number of goblet cells in chitosan-treated groups in a dose-dependent manner (Table 2). Additionally, there was a positive correlation between the thickness of the enterocyte brush border and the dose of chitosan mediating the increase in absorptive activity.

**Figure 1:**
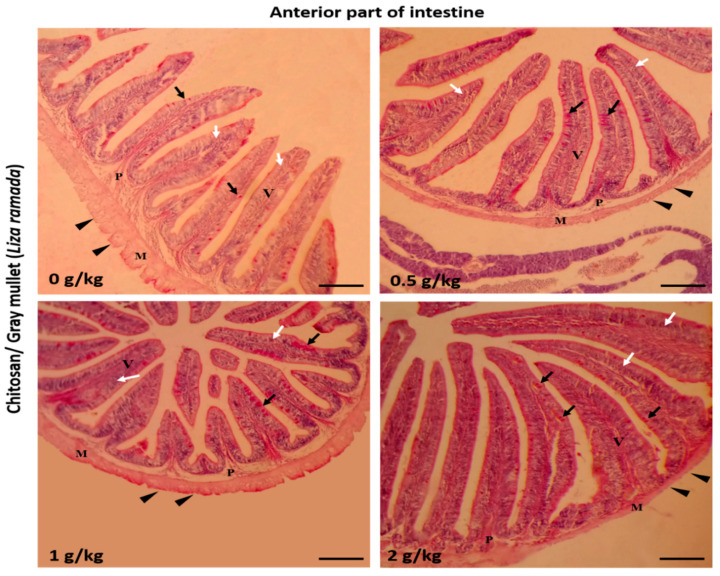
Histomicrograph showing the histological structures of the anterior part of Grey Mullet intestines in the control group and chitosan-fed groups (0.5, 1, and 2 g/kg). The intestine shows normal histological structures of the intestinal wall and intestinal villi in all groups. The intestinal wall was formed of tunica mucosa of normally arranged enterocyte (white arrow) and goblet cells (black arrow), propria submucosa (P), tunica muscularis (M), and tunica serosa (black arrowhead). The villous height, width, and the number of goblet cells increased gradually in a dose-dependent manner with chitosan. Stain periodic acid-Schiff (PAS). Bar = 200 µm.

**Figure 2:**
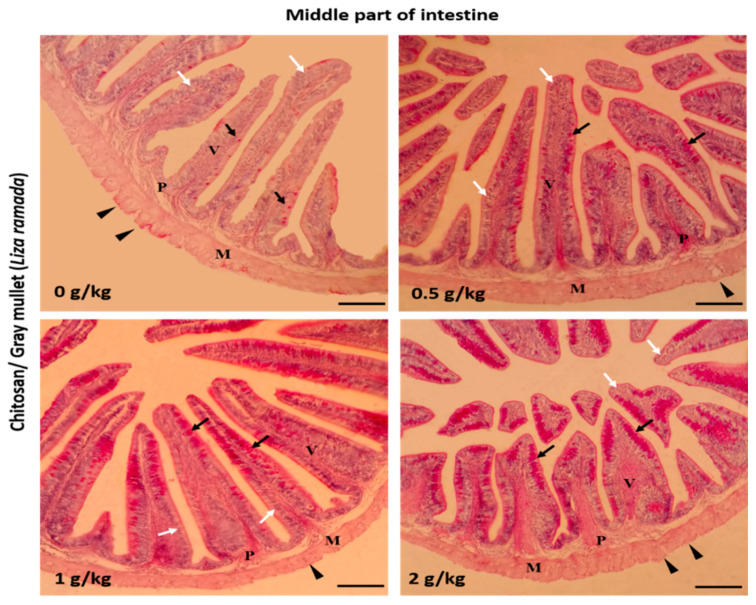
Histomicrograph showing the histological structure of the middle part of Grey Mullet intestines in the control group as well as chitosan-fed groups (0.5, 1, and 2 g/kg). The intestine shows normal histological structures of the intestinal wall and intestinal villi in all groups. The intestinal wall was formed of tunica mucosa of normally arranged enterocyte (white arrow) and goblet cells (black arrow), propria submucosa (P), tunica muscularis (M), and tunica serosa (black arrowhead). The goblet cells were more prominent and gave a positive periodic acid-Schiff (PAS) reaction. The villous height, width, and the number of goblet cells increased gradually in a dose-dependent manner with chitosan. Stain PAS. Bar = 200 µm.

**Figure 3:**
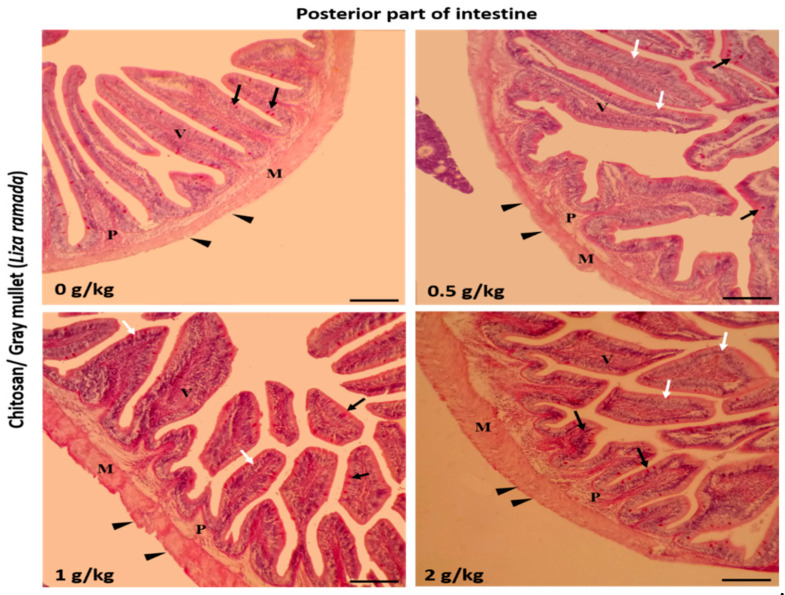
Histomicrograph showing the histological structure of the posterior part of Grey Mullet intestines in the control group and chitosan-fed groups (0.5, 1, and 2 g/kg). The intestine shows normal histological structures of the intestinal wall and intestinal villi in all groups. The intestinal wall was formed of tunica mucosa of normally arranged enterocyte (white arrow) and goblet cells (black arrow), propria submucosa (P), tunica muscularis (M), and tunica serosa (black arrowhead). The villous height and width increased gradually in a dose-dependent manner with chitosan. Stain periodic acid-Schiff (PAS). Bar = 200 µm.

Histologically, the intestinal walls of Grey Mullet consist of four layers; mucosa, sub-mucosa, tunica muscularis (muscular layers), and serosa. The histological examination of the *L. ramada* intestine showed a normal histo-morphology. The epithelial layer of the intestinal mucosa was thrown into elongated finger-like projections, the intestinal villi. The epithelial lining of the intestinal villi was made of the absorptive columnar epithelium (enterocyte) and mucous-secreting goblet cells, as demonstrated using periodic acid-Schiff (PAS) staining. The brush borders of enterocytes were PAS-positive and appeared in the treated groups. The intestinal villi and associated crypt appeared free of any inflammatory or degenerative changes. Furthermore, the enterocytes and goblet cells were properly arranged (Figure 1, Figure 2 and Figure 3).

### 2.3. Haemato-Biochemical Indices

The values of hemoglobin, hematocrit, red blood cells (RBCs), total protein (TP), albumin, and globulin were significantly increased in fish fed 1 and 2 g/kg of chitosan nanoparticles compared to fish fed 0 and 0.5 g/kg (*p* < 0.05) (Table 3 and Table 4). The highest levels of TP and albumin were observed in fish fed 1 g/kg diet (*p* < 0.05). 

### 2.4. Immune Response

**Figure 4:**
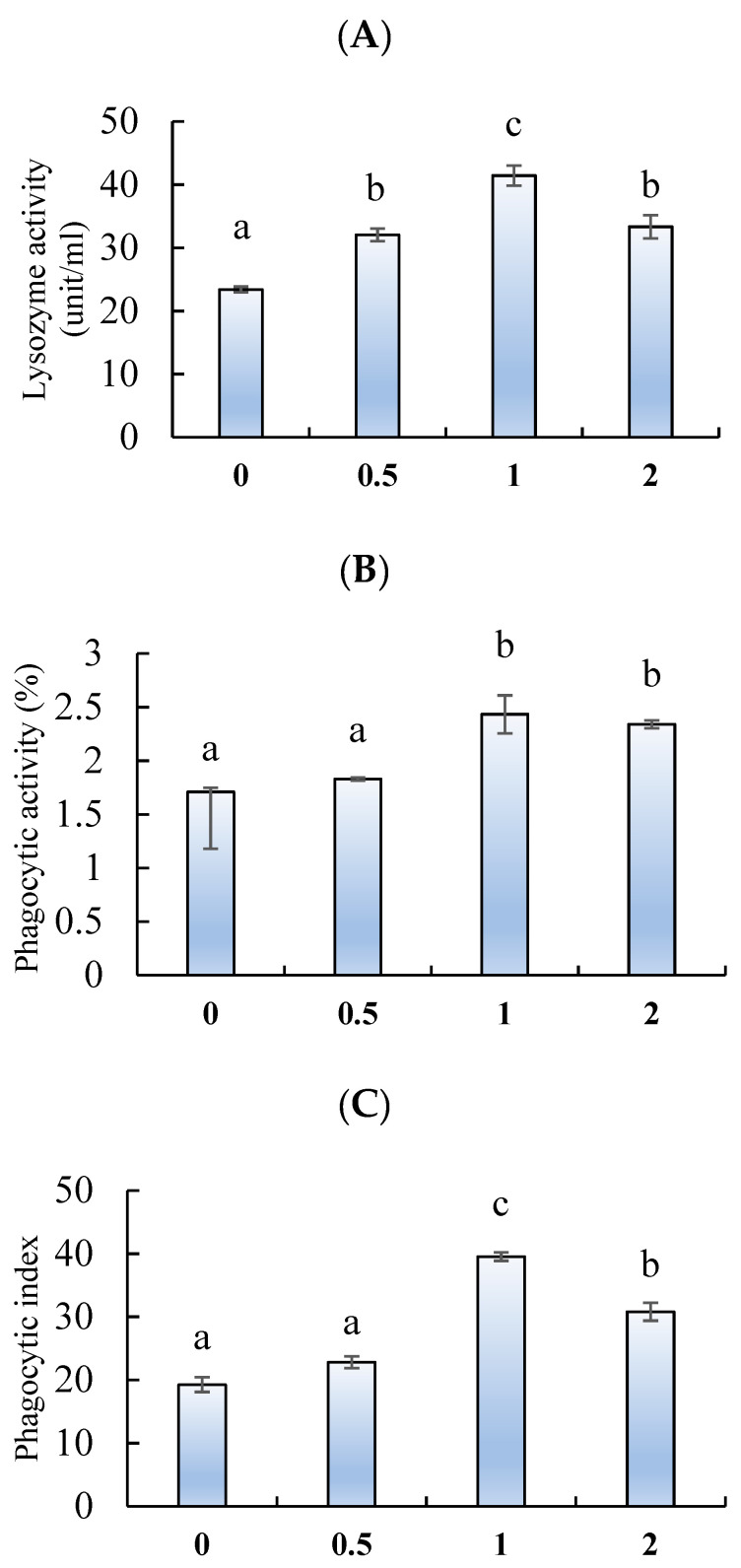
(**A**) Lysozyme activity, (**B**) phagocytic activity, and (**C**) phagocytic index of *L. ramada* fed varying levels of chitosan nanoparticles. Bars with different letters indicate significant differences among the groups (as mean ± SE, n = 3).

The lysozyme activity (Figure 4A) and phagocytic index (Figure 4B) were significantly enhanced by feeding chitosan nanoparticles at 0.5, 1, and 2 g/kg, whereas the phagocytic activity (Figure 4C) was improved in fish fed 1 and 2 g/kg (*p* < 0.05). The highest lysozyme activity and phagocytic index were observed in fish fed 1 g/kg.

### 2.5. Anti-oxidative Response

**Figure 5:**
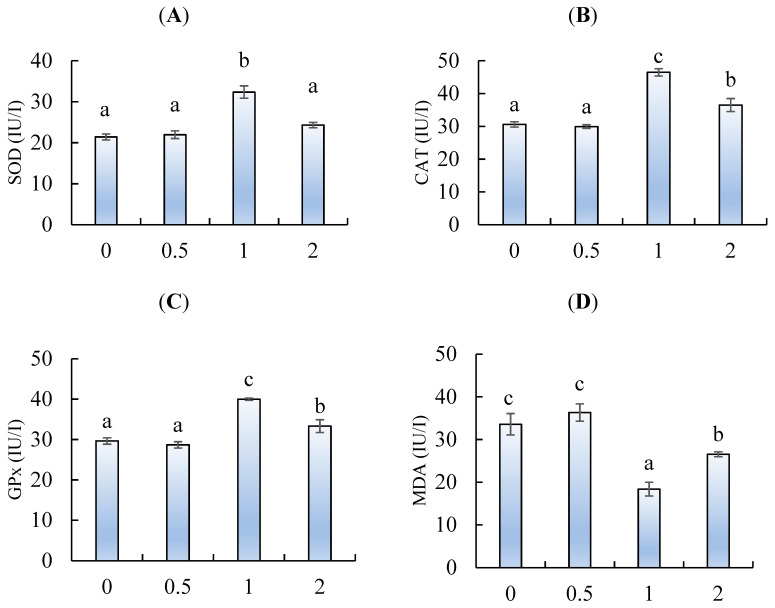
(**A**) Superoxide dismutase (SOD), (**B**) catalase (CAT), (**C**) glutathione peroxidase, and (**D**) malondialdehyde (MDA) of *L. ramada* fed varying levels of chitosan nanoparticles. Bars with different letters indicate significant differences among the groups (as mean ± SE, n = 3).

Viewing Figure 5A, the superoxide dismutase (SOD) was significantly activated by feeding chitosan nanoparticles at 1 g/kg. The glutathione peroxidase (GPx) (Figure 5B) and catalase (CAT) (Figure 5C) activities also were enhanced by feeding chitosan at 1 and 2 g/kg, when compared to fish fed 0 and 0.5 g/kg (*p* < 0.05). The highest GPx and CAT activities were observed in fish fed 1 g/kg (*p* < 0.05). Conversely, the levels of malondialdehyde (MDA) were decreased by feeding chitosan at 1 and 2 g/kg, with the lowest being in fish fed 1 g/kg (Figure 5 D) (*p* < 0.05).

## 3. Discussion

Marine-derived chitosan has diverse biological influences on the performance of aquatic animals [10,15]. Chitosan can be extracted from crustacean exoskeletons and structured through the deacetylation of chitin with abundant amounts of copolymers of glucosamine N-acetylglucosamine, which are stable during digestion and can tolerate the hydrolysis function of the digestive enzymes [25]. Correspondingly, chitosan can stay stable for a long time until it can reach the targeted organ. Further, the nanoparticle form of chitosan facilitates its functionality and absorption at low supplementary levels [24]. As expected, the results clearly show an enhanced growth performance and feed utilization of *L. ramada* fed dietary chitosan nanoparticles, which can be attributed to the potential role of chitosan in improving the intestinal-histo-morphometric features. The inclusion of dietary chitosan at the rate of 1–2 g/kg resulted in the best growth rate and histomorphometrical features for the intestine of *L. ramada*. Concurrently, the absorption of nutrients by the intestinal villi was enhanced and the natural metabolic functions were regulated, which resulting in improved blood health, immune response, and anti-oxidative responses. These results illustrate that the inclusion of chitosan nanoparticles is recommended in a dose-dependent manner to improve aquatic animal performances and relieve the expected inflammation that can result from stressful aquaculture practices.

The results of the current study showed enhanced growth performance of *L. ramada* fed diets with chitosan nanoparticles. Similarly, the growth performance of Nile tilapia [19,20,21,22], rainbow trout [23], silver carp [24], and loach [15] was improved by dietary chitosan. The growth-promoting activity of chitosan is attributed to its role in activating the digestive enzymes by inhibiting the pathogenic bacteria and the activation of beneficial ones [26]. Accordingly, the beneficial microorganisms can secrete digestive enzymes to digest the feed and convert it to available nutrients for absorption through the intestinal villi [27]. The antibacterial capacity of chitosan reduces the abundance of harmful microorganisms in the fish intestines and, in turn, enhances the local intestinal immunity [9]. The obtained results also showed improved feed utilization (reduced feed conversion ratio) in *L. ramada* fed chitosan nanoparticles. The enhanced feed efficacy also explains the improved growth performance in the present study. Although the digestive enzyme activity was not detected in the present study, the inclusion of chitosan nanoparticles was proved to activate the intestinal digestive enzymes in other fish species (e.g., Nile tilapia [19,22]). The enhancement of digestive enzyme activity also could explain the potential role of chitosan nanoparticles in improving the feed utilization and the growth performance of *L. ramada* Zhang [28] attributed the enhanced growth rate and feed utilization to chitosan’s role in activating digestive enzymes, which are responsible for the hydrolysis of nutrients (e.g., carbohydrates, lipids, and proteins) in the gastrointestinal tract (GIT). Correspondingly, the digested nutrients can be easily absorbed by the intestinal villi and be available for the cells. 

The measurement of intestinal histomorphometrical features is a vital tool for evaluating the effect of dietary supplements on the intestinal absorption capacity and the neighboring intestinal immunity, as well as its relationship to the general immune system in the fish body [6,29]. The main findings of the current investigation are the enhancement of the intestinal morphometrical and histological features in *L. ramada* fed chitosan nanoparticles. The intestinal villi and width were improved, while the number of goblet cells were increased in fish treated with chitosan nanoparticles. Concurring, Abd El-Naby et al. [22] reported that Nile tilapia fed chitosan nanoparticles had improved intestinal histological features. The improved intestinal morphometry refers to the increased surface of intestinal villi to absorb the digested nutrients in the intestine [30]. The enhanced intestinal health and morphometry indices explain the enhanced feed utilization in *L. ramada* fed dietary chitosan nanoparticles. Dietary chitosan nanoparticles enhanced the diversity and integrity of the intestinal epithelial cells which, correspondingly, alleviated the intestinal pathogenicity and inflammation that can be attributed to the antibacterial effect of chitosan. The results are in line with [31], who stated that chitosan improved the local intestinal integrity and immunity of Pacific white shrimp (*Litopenaeus vannamei*). Concurrently, the enhanced local immunity led to enhanced humoral and innate immune responses due to chitosan feeding.

The suitability of feed supplements also can be assessed using hematological parameters, such as red blood cells (RBCs), white blood cells (WBCs), hemoglobin concentrations, and hematocrit levels [32]. Changes in the values of these parameters may occur due to the fish’s health condition and nutritional behavior [33]. Notably, the results show that *L. ramada* fed chitosan nanoparticles increased WBCs, RBCs, hemoglobin, and hematocrit values. These results confirm the safe use of chitosan nanoparticles and their beneficial influence on the health condition of *L. ramada*. Concurrently, Abd El-Naby et al. [22] and Younus et al. [24] illuminated that Nile tilapia and silver carp fed chitosan nanoparticles had increased WBCs, RBCs, hemoglobin, and hematocrit values, and attributed this influence on the potential role of chitosan in enhancing the antibacterial, immunity and anti-oxidative conditions.

Lysozyme activity is a part of the immune system that can deactivate the pathogenicity of the harmful bacteria in the fish body [34]. Likewise, phagocytosis is a function where phagocytic cells can attack the pathogenic bacteria and resist its pathogenic role on fish [35]. Marine-derived polysaccharides are known for their immunomodulation influence and anti-inflammatory and antibacterial capacities [10]. Relatedly, this study’s results clearly show improved lysozyme and phagocytic activities in *L. ramada* fed dietary chitosan nanoparticles. These results indicate that *L. ramada* has a resistance against the common infection attributed to pathogens in fishponds. Paralleling our study, Younus et al. [24] and Khani Oushani et al. [23] illustrated that silver carp and rainbow trout fed dietary chitosan nanoparticles had enhanced lysozyme and antibacterial activity. Interestingly, the enhanced phagocytosis activity seen in the present study accompanies the increased number of WBCs, which explains the improved immune response of *L. ramada* fed dietary chitosan nanoparticles. Victor et al. [36] suggested that chitosan can activate a fish’s immunity by regulating innate immunity through Toll-like receptor-regulated signaling pathways. However, further studies are recommended to clarify the mode of action that allows chitosan to enhance aquatic animal immune responses.

Oxidative stress is caused by an imbalance in the production of reactive oxygen species (ROS) and antioxidant enzymes [37]. Fish have an anti-oxidative defense mechanism for ROS [38]. This defense mechanism includes antioxidant enzymes (e.g., superoxide dismutase (SOD), catalase (CAT), and glutathione peroxidase (GPx)) that can inhibit ROS production and protect the body from damage such as deoxyribonucleic acid (DNA) strand breaks, lipid peroxidation, protein oxidation, plus the impairment of the cell metabolism function and structure [39]. Lipid peroxidation occurring in the cells can be measured by the concentration of malondialdehyde (MDA) [40]. Considering aquaculture, chitosan is frequently used to prepare a fish feed and has been confirmed to present anti-oxidative activity when administered orally to fish [13]. During the present study, *L. ramada* fed dietary chitosan nanoparticles had enhanced SOD, CAT, and GPx and reduced MDA levels. Similarly, Nile tilapia [22] and loach [15] fed dietary chitosan nanoparticles showed an enhanced anti-oxidative capacity. Chitosan nanoparticles enhanced the antioxidant ability probably due its chelating ability and the scavenging role of free radicals [41]. The results suggest that dietary chitosan resulted in enhanced SOD, CAT, and GPx neutralizing oxidative stress-induced damage, such as lipid peroxidation (MDA) [42] that may occur during the farming season of *L. ramada*. It has been hypothesized that dietary chitosan probably activates the anti-oxidative response by improving feeding utilization, allowing dietary antioxidants to play a role in antioxidant activity [42]. More specifically, chitosan’s anti-oxidative capacity overcomes oxidative stress, probably induced by harmful microorganisms, in the GIT of fish.

The results show that fish fed chitosan nanoparticles at 2 g/kg diet had relatively lower immune and anti-oxidative responses than fish fed 1 g/kg. These results are probably due to the necessity of including chitosan nanoparticles in a dose-dependent manner. The inclusion of functional feed additives is known for the species-specific doses depending on the fish species, feeding duration, and rearing conditions [5]. The results also confirm the proposed hypothesis that the nano form of chitosan could be included in lower levels compared to organic and other forms [43]. Few studies in the literature explain the role of chitosan in improving the performances of aquatic animals. However, no investigations to our knowledge were done to apply the mechanistic role (e.g., quantitative real-time polymerase chain reaction (Q-PCR) and Western Blot) of chitosan in aquatic animals. Although the study limits the application of related genetic analysis that can explain chitosan functionality in improving the histomorphometrical features, the histological study conducted in the present study opens the door for further future studies that explain the mechanistic effect of chitosan in enhancing the performance of aquatic animals.

## 4. Materials and Methods

### 4.1. Ethical Approval

The protocol of the present experiment and fish rearing techniques were approved and conducted with the guidance of the Research Ethical Committee, Faculty of Agriculture, Kafrelsheikh University, Egypt.

### 4.2. Preparation of Chitosan Nanoparticles

**Figure 6:**
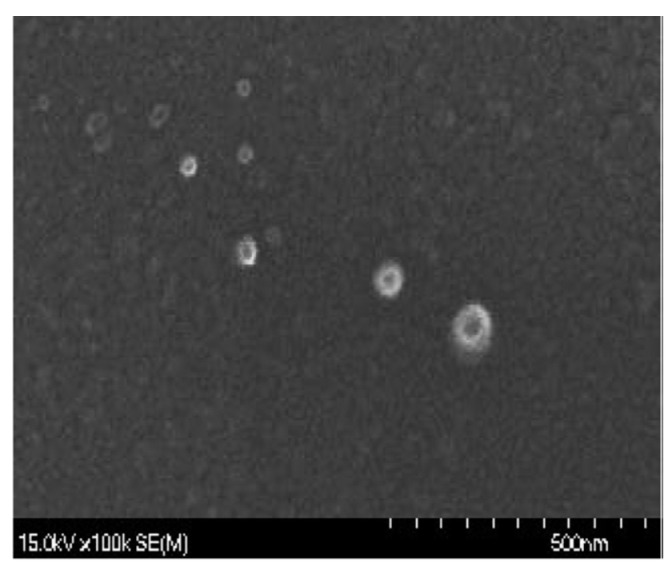
The morphological feature of chitosan nanoparticles observed by a scanning electron microscope (SEM).

Chitosan nanoparticles were prepared by following Tang, et al. [44] with some modifications. Chitosan was dissolved in 80 mL distilled water containing 1% glacial acetic acid to turn the pH acidic, then magnetically stirred for 30 min to fully dissolve. Then, 20 mL of sodium tripolyphosphate (TPP) solution was dropped into the chitosan beaker at room temperature while stirring. Subsequently, the chitosan solution was magnetically stirred for 45 mins to obtain chitosan nanoparticles (Figure 6). These chitosan nanoparticles could be stably stored at −8 °C.

### 4.3. Fish, Design, and Diets

A stock of visually similar sizes of Grey Mullet (*L. ramada*) juveniles was collected from a local farm in Kafr El-Sheikh governorate and gently transported to the laboratory to avoid stressful transportation effects on the fish. Fish were then stocked in a concrete tank (5 × 10 × 1 m), which provided continuous aeration, and kept for ten days for adaptation. During the acclimatization period, fish were fed the control diet, and half of the water was replaced daily with freshly dechlorinated water. Then, fish with similar average initial weights (52.95 ± 0.81 g) were randomly selected and distributed in 12 concrete tanks (2 × 5 × 1 m) (4 groups/3 replicates), wherein each tank were placed 50 fish. The tanks were in an outdoor area under the conditions of a flow-through system. During the trial (8 weeks), the water characteristics were checked regularly and reported to be within the normal values for the optimal growth of common carp, and experienced a photoperiod (12 h light: 12 h darkness). The water temperature, pH, dissolved oxygen, and total ammonia averaged 25.34 ± 0.22 °C, 7.62 ± 0.54, 6.11 ± 0.31 mg/L, and 0.02 ± 0.001 mg/L, respectively.

The basal diet was formulated by well-mixing fish meal, soybean meal, yellow corn, wheat bran, rice bran, and a vitamin and mineral mixture (Table 5). Then, pellets were prepared with fish oil and water to prepare a dough of 1–2 mm in size using the laboratory pelletizer. Four types of diets were prepared, where the control diet was not supplemented with chitosan nanoparticles and the second, third, and fourth diets were supplemented with chitosan nanoparticles at 0.5, 1, and 2 g/kg, respectively. The prepared diets were then dried at room temperature and kept at 4 °C. Fish were fed the diets at 2–3% throughout the trial.

### 4.4. Final Sampling

Upon conclusion of the trial, all fish fasted 24 hours before the final sampling. Then, fish in each tank were weighed and counted to calculate the growth performance, feed efficiency, and survival indices. The following equations were used for the calculation: Weight gain (WG, %) = (FBW − IBW) × 100/IBW, specific growth rate (SGR, %g/day) = 100((LnFBW − LnIBW)/T), feed conversion ratio (FCR) = FI/WG, and survival (%) = (final NO./initial NO.) ×100, where FBW = body weight final (g), IBW = body weight initial (g), T = duration (days), FI = feed intake (g), and NO. = number of fish.

Afterward, fish were anesthetized (100 mg/L tricaine methanesulfonate), and three fish from each tank were randomly collected for blood sampling and dissection. Fish were bled from the caudal vein with a nonheparinized syringe for serum collection (3000 rpm for 15 min at 4 °C), while heparinized syringes were used for the whole blood collection. The whole blood was used for the hematological and phagocytic analysis, while the collected serum was kept at −80 °C until use. Then, fish were gently dissected and the intestines were collected and rinsed in Boin’s solution for histology sections.

### 4.5. Blood Analysis

Red blood cells (RBCs) and white blood cells (WBCs) were counted with a hemocytometer immediately after dilution with Natt and Herrick’s solution [46]. Regarding the differential leucocytic count, blood films were prepared and stained according to Lucky [47], while cells were calculated by following Jain [48]. Blood hemoglobin concentration was determined with a spectrophotometer (Model RA 1000, Technicon Instruments Corporation Tarrytown, NY USA) at 540 nm using the Blaxhall and Daisley [49] method.

According to Doumas, et al. [50] and Dumas [51], the serum total protein and albumin were determined. The globulin content was calculated mathematically. The activities of aspartate aminotransferase (AST) and alanine aminotransferase (ALT) were determined calorimetrically at a wavelength of 540 nm [52]. Serum creatinine and urea were calorimetrically determined according to Coulombe and Favreau [53] and Heinegård and Tiderström [54], respectively.

Blood lysozyme activity was determined using the turbidimetric assay [55]. The phagocytic activity and phagocytic index were determined according to Kawahara, et al. [56].

Superoxide dismutase (SOD), catalase (CAT), and glutathione peroxidase (GPx) in serum were measured using diagnostic reagent kits following the manufacturer’s (Cusabio Biotech Co., Ltd.; China) instructions. The detailed instructions for SOD, CAT, and GPx measurements are written in Catalog Numbers: CSB-E15929Fh, CSB-E15928Fh, and CSB-E15930Fh, respectively. Briefly, the reagents and samples were prepared according to the instructions then the blank was set well and 50 μL of SOD, CAT, and GPx standards or samples were added to each well. Fifty microliters of Horseradish Peroxidase (HRP) conjugate (1X) was added to each well except the blank one, then incubated at 37 °C for one hour, aspirated and washed five times, then 90 μL of TMB (3,3’,5,5’-Tetramethylbenzidine) substrate was added to each well and incubated in a dark place at 37 °C for half an hour. Subsequently, 50 μL of stop solution was added to each well and read at 450 nm within 10 minutes. 

Serum malonaldehyde (MDA) content was assessed, according to Ledwozyw et al. [57]. One mL of serum was mixed with 2 mL of trichloroacetic acid (TBA) and hydrochloric acid under acidic circumstances then the mixture was diluted to two hundred mL using water and kept for half an hour. It was then cooled and centrifuged at 300 rpm for 10 minutes, the supernatant was separated, and the absorption was measured at 535 nm.

### 4.6. Intestinal Morphometry

The histological investigation was carried out at the end of the experiment. Nine Grey Mullet fish were randomly selected for each treatment (3 fish from each aquarium). After deep anesthesia using 40% ethyl alcohol, the abdomen was dissected and specimens from the anterior, middle, and terminal parts of the intestine were sampled. The samples were immediately fixed for 48 h in 10% neutral buffered formalin solution. After fixation, tissue specimens were processed according to Gewaily et al. [58]. Briefly, the tissue samples were dehydrated in ascending grades of ethyl alcohol, cleared in xylene, embedded in paraffin wax, cut into several 5 μm thick sections using a rotary microtome (RM 20352035; Leica Microsystems, Wetzlar, Germany), and mounted on clean slides. The paraffin sections were rehydrated and stained with periodic acid-Schiff (PAS) according to the methodology described by Bancroft and Gamble [59] for general morphometry and goblet cell identification. Afterward, several representative photomicrographs were captured from the stained sections with a digital camera (Leica EC3, Leica, GmbH, Wetzlar, Germany) connected to a microscope (Leica DM500).

The morphometric study was adopted by examining villus height (μm, from the tip to the base of villus), villus width (μm, at the mid-height of villi), and the number of PAS-positive goblet cells. The measurements were done using a computerized image analysis system (Image J software; Bethesda, MD, USA) [60]. The results were revealed as means ± standard error (SE). We used the statistical software package SPSS 22 (SPSS^®^ Inc., Chicago, IL, USA) to perform the statistical analysis. 

### 4.7. Statistical Analysis

Normality and homoscedasticity analyses were established using Shapiro-Wilk and Levene tests before applying a one-way ANOVA method to confirm the normal distribution of the data, then the obtained data were subjected to a one-way ANOVA using SPSS version 22 (SPSS^®^ Inc., Chicago, IL, USA). Data were presented as means ± the standard error (SE). Differences between the means were tested at the 5% probability level using a Duncan test as a post-hoc test.

## 5. Conclusions

To summarize, the results elucidated that *L. ramada* fed dietary chitosan nanoparticles had a marked growth performance and feed utilization. These improvements were attributed to the potential role of chitosan nanoparticles in enhancing the intestinal histo-morphometry and antibacterial capacity. The immunity and anti-oxidative responses also were improved with the continuous feeding of chitosan nanoparticles. These results soundly supported the possibility of using chitosan nanoparticles as a feasible functional supplement for aquatic animals. 

## Figures and Tables

**Table 1:** Growth performance and nutrient utilization of *L. ramada* fed varying levels of chitosan nanoparticles *.

Item	Chitosan Nanoparticles (g/kg)			
	0	0.5	1	2
IBW (g)	52.95 ± 0.05	52.91 ± 0.13	52.95 ± 0.05	53.00 ± 0.00
FBW (g)	106.26 ± 0.02 ^a^	107.33 ± 0.34 ^b^	107.83 ± 0.13 ^b^	108.77 ± 0.17 ^b^
WG (%)	100.67 ± 0.15 ^a^	102.88 ± 1.01 ^b^	103.63 ± 0.39 ^b^	105.23 ± 0.33 ^b^
SGR (%/day)	1.16 ± 0.00 ^a^	1.18 ± 0.01 ^b^	1.19 ± 0.00 ^b^	1.20 ± 0.00 ^b^
FCR	1.78 ± 0.00 ^c^	1.72 ± 0.03 ^b^	1.69 ± 0.01 ^b^	1.63 ± 0.02 ^a^
Survival (%)	95.24 ± 0.95	99.05 ± 0.95	98.10 ± 1.90	97.14 ± 1.65

* Different superscripts refer to differences between all groups for each parameter (*p* < 0.05). IBW: initial body weight; FBW: final body weight; WG: weight gain; SGR: specific growth rate; FCR: feed conversion ratio.

**Table 2:** Intestinal morphometry indices of *L. ramada* fed varying levels of chitosan nanoparticles *.

Item	Chitosan Nanoparticles (g/kg)			
	0	0.5	1	2
Anterior intestine				
Villi height (μm)	410.00 ± 18.63 ^a^	414.32 ± 7.10 ^a^	439.26 ± 9.36 ^a^	623.04 ± 12.63 ^b^
Villi width (μm)	70.70 ± 6.68 ^a^	86.05 ± 4.33 ^b^	93.17 ± 1.76 ^bc^	106.67 ± 5.71 ^c^
Goblet cells /mm^2^	5.20 ± 0.86 ^a^	8.60 ± 0.75 ^b^	10.60 ± 0.60 ^b^	10.80 ± 0.37 ^b^
Middle intestine				
Villi height (μm)	383.53 ± 33.80 ^a^	410.55 ± 16.55 ^a^	484.38 ± 10.33 ^b^	498.83 ± 4.77 ^b^
Villi width (μm)	76.43 ± 1.98 ^a^	81.80 ± 4.42 ^a^	108.22 ± 3.70 ^b^	124.02 ± 10.44 ^b^
Goblet cells /mm^2^	4.80 ± 0.66 ^a^	11.80 ± 1.07 ^b^	20.80 ± 2.27 ^c^	23.20 ± 1.56 ^c^
Posterior intestine				
Villi height (μm)	395.45 ± 15.71 ^a^	447.51 ± 12.48 ^ab^	467.52 ± 20.98 ^b^	537.87 ± 13.72 ^c^
Villi width (μm)	77.85 ± 7.09 ^a^	97.50 ± 2.86 ^b^	112.78 ± 6.98 ^bc^	127.76 ± 10.01 ^c^
Goblet cells /mm^2^	3.80 ± 0.37 ^a^	6.80 ± 0.58 ^b^	8.40 ± 0.51 ^c^	9.60 ± 0.40 ^c^

* Different superscripts refer to differences between all groups for each parameter (*p* < 0.05).

**Table 3:** Hematological indices of *L. ramada* fed varying levels of chitosan nanoparticles *.

Item	Chitosan Nanoparticles (g/kg)			
	0	0.5	1	2
Hb (g/100ml)	7.50 ± 0.15 ^a^	7.37 ± 0.12 ^a^	8.53 ± 0.20 ^b^	8.16 ± 0.10 ^b^
RBCs (10/mm^6^)	2.50 ± 0.05 ^a^	2.46 ± 0.04 ^a^	2.84 ± 0.07 ^b^	2.72 ± 0.04 ^b^
PCV (%)	24.25 ± 0.46 ^a^	23.90 ± 0.38 ^a^	27.58 ± 0.65 ^b^	26.42 ± 0.34 ^b^
MCV (mm^3^)	97.00 ± 0.01	97.14 ± 0.14	97.00 ± 0.01	97.00 ± 0.01
MCH (Pg)	30.01 ± 0.03	29.96 ± 0.05	30.00 ± 0.02	29.98 ± 0.01
MCHC (%)	30.94 ± 0.03	30.84 ± 0.02	30.93 ± 0.02	30.90 ± 0.01
WBCs (10/mm^3^)	39.18 ± 0.60	39.16 ± 1.17	39.44 ± 0.39	39.07 ± 0.44
Heterophils (%)	10.67 ± 0.33	10.33 ± 0.33	11.00 ± 0.58	10.33 ± 0.33
Lymphocytes (%)	80.00 ± 0.58	80.00 ± 0.58	79.67 ± 0.67	80.00 ± 0.00
Monocytes (%)	6.67 ± 0.33	7.33 ± 0.33	7.33 ± 0.33	7.00 ± 0.00
Eosinophils (%)	1.67 ± 0.33	1.67 ± 0.33	1.33 ± 0.33	2.00 ± 0.00
Basophils (%)	1.00 ± 0.00	0.67 ± 0.33	0.67 ± 0.33	0.67 ± 0.33

* Different superscripts refer to differences between all groups for each parameter (*p* < 0.05). Hb: hemoglobin; RBCs: red blood cells; PCV: packed cell volume; MCV: mean corpuscular volume; MCH: mean corpuscular hemoglobin; MCHC: mean corpuscular hemoglobin concentration; WBCs: white blood cells.

**Table 4:** Blood biochemical indices of *L. ramada* fed varying levels of chitosan nanoparticles *.

Item	Chitosan Nanoparticles (g/kg)			
	0	0.5	1	2
ALT (U/I)	3.27 ± 0.03	3.11 ± 0.06	2.69 ± 0.02	2.88 ± 0.05
AST (U/I)	74.70 ± 2.24	72.30 ± 1.41	64.97 ± 1.97	72.97 ± 2.11
ALP (U/I)	84.33 ± 1.40	83.37 ± 1.04	73.00 ± 2.14	79.43 ± 1.45
Total protein (g/dl)	3.06 ± 0.04 ^a^	2.95 ± 0.03 ^a^	4.13 ± 0.04 ^c^	3.54 ± 0.04 ^b^
Albumin (g/dl)	1.27 ± 0.01 ^a^	1.22 ± 0.01 ^a^	1.77 ± 0.01 ^c^	1.34 ± 0.01 ^b^
Globulin (g/dl)	1.79 ± 0.02 ^a^	1.73 ± 0.02 ^a^	2.36 ± 0.10 ^b^	2.21 ± 0.04 ^b^
Urea (mg/dl)	3.90 ± 0.04	3.98 ± 0.07	3.52 ± 0.01	3.85 ± 0.02
Creatinine (mg/dl)	0.23 ± 0.01	0.26 ± 0.01	0.19 ± 0.00	0.22 ± 0.01

* Different superscripts refer to differences between all groups for each parameter (*p* < 0.05). ALT: alanine aminotransferase; AST: aspartate aminotransferase; ALP: alkaline phosphatase.

**Table 5:** Basal diet and proximate chemical composition (%, on dry matter basis).

Ingredients	%	Chemical Composition	%
Fish meal	15	Crude protein	34.49
Soybean meal	40	Crude lipids	6.29
Yellow corn	15	Ash	7.55
Gluten	7	Fibers	5.12
Wheat bran	12	Gross energy (kcal/kg) ^2^	1863.10
Wheat flour	4.92		
Fish oil	3		
Vitamin and mineral mix ^1^	2		
Dicalcium phosphate	1		
Vitamin C	0.08		

^1^ Vitamin and mineral mixture detailed by Dawood et al. [45]. ^2^ Gross energy was calculated based on protein, lipid, and carbohydrate values as 23.6, 39.5, and 17.2 KJ/g, respectively.
